# Associations between dairy consumption, physical activity, and blood pressure in Chinese young women

**DOI:** 10.3389/fnut.2023.1013503

**Published:** 2023-04-11

**Authors:** Yining Lu, Huw D. Wiltshire, Julien S. Baker, Qiaojun Wang, Shanshan Ying

**Affiliations:** ^1^Faculty of Sport Science, Ningbo University, Ningbo, China; ^2^Cardiff School of Sport and Health Sciences, Cardiff Metropolitan University, Cardiff, United Kingdom; ^3^Centre for Population Health and Medical Informatics, Department of Sport, Physical Education and Health, Hong Kong Baptist University, Kowloon Tong, Hong Kong SAR, China

**Keywords:** dairy intake, physical activity, accelerometer, blood pressure, young women

## Abstract

**Introduction:**

The prevalence of hypertension (HTN) has been increasing in young adults. A healthy dietary pattern and increasing physical activity (PA) are commonly recommended as lifestyle modifications needed to manage blood pressure (BP). However, little is known about the relationship between dairy intake, PA, and BP in Chinese young women. The aim of this study was to examine whether BP was associated with dairy intake, moderate-to-vigorous intensity physical activity (MVPA) and total physical activity (TPA) in a sample of Chinese young women.

**Methods:**

A total of 122 women (20.4 ± 1.4) who had complete data sets from the Physical Fitness in Campus (PFIC) study were included in this cross-sectional analysis. Data related to dairy intake and PA was collected using a food frequency questionnaire and an accelerometer. BP was measured following standardized procedures. The association between BP with dairy intake and PA was examined using multivariable linear regression models.

**Results:**

After controlling for potential covariables, we observed a significant and independent relationship only between systolic BP with dairy intake [standardized beta (b) = −0.275, *p* < 0.001], MVPA (*b* = −0.167, *p* = 0.027), and TPA (*b* = −0.233, *p* = 0.002). Furthermore, we found a decrease of 5.82 ± 2.94, 1.13 ± 1.01, and 1.10 ± 0.60 mm Hg in systolic BP for daily additional servings of dairy, 10 min of MVPA, and 100 counts per minute of TPA, respectively.

**Conclusion:**

Our results suggested that the higher amount of dairy consumption or PA was associated with lower level of SBP in Chinese young women.

## Introduction

The prevalence of hypertension (HTN) has emerged as one of the main risk factors for cardiovascular disease (CVD) worldwide (1). Although high blood pressure (BP) is generally observed among elderly populations, epidemiological research has reported an increase in the incidence of elevated BP in young adults (2, 3). A recent meta-analysis including 4.5 million young adults revealed that young adults with high BP are more likely to develop CVD in later life (4). Moreover, an association between raised BP in young adulthood and the increased risk of premature CVDs has also been observed (5) and this association is independent of later adult exposures (6). There has been a substantial increase in HTN-related CVDs burden in young adults from 1990 to 2019 globally (7), therefore, promoting an effective HTN management method is essential.

Poor dietary habits and physical inactivity are well documented as traditional risk factors for elevated BP (2). Scientific evidence from randomized controlled trials and prospective cohort studies has demonstrated beneficial effects, including the Dietary Approaches to Stop Hypertension (DASH) diet on the control of BP in middle-aged and older adults, (8–11). Dairy products, included as one of the components of the DASH eating pattern, have not been investigated fully in relation to beneficial effects on the BP of young women. Due to the effects that sex hormones have on BP in relation to estrogens having a protective effect on BP while androgens have a hypertensive effect (12), some studies suggest that these hormones result in differences in effects of dairy intake on the occurrence of HTN and that women are more likely to benefit from dairy intake (13, 14). Furthermore, a favorable association between dairy consumption and BP in young women has been observed previously (15, 16). However, these findings are derived from Western experimentation and subjects. Since the pattern and the amount of dairy consumption varies between countries and ethnicities, there are geographical, regional, and ethnicity-related differences in the association between dairy intake and HTN. A recent systematic review has shown reduced BP with dairy intake among Americans, while no effects were recorded in Asian populations (14). DellaValle et al. (17) reported an inverse relationship between dairy intake and SBP in white children, but not in black children. In China, the average dairy consumption increased from 25 g in 2012 to 40 g in 2021, which is still much lower than dairy consumption in Western populations (18). In addition, this increase is still lower than the recommended daily intake of 300 g outlined in the 2016 Chinese dietary guideline (19). Despite the low level of dairy intake, some studies observed an inverse relationship with BP in older Chinese (20, 21), however, data for Chinese young women is still minimal.

Also, despite the evidence strongly supporting a BP reducing effect from physical activity (PA) for adults (22–25), this positive effect is undetectable in several studies based on adult women exclusively (26–29). Since this experimental evidence is derived from Western populations, including Caucasian, Hispanic, Pacific, and Latina women, limited data are available for Chinese women.

Physical activity and dietary behaviors demonstrate some correlations. For example, physically active individuals tend to consume more healthy food and nutrients (e.g., fruits, vegetables, fiber, calcium, and vitamins) than their less active counterparts (30); PA was associated with lower sugar-sweetened beverages intake among adolescent boys (31). Although several studies have investigated the effects of individual behaviors such as diet and PA, little research has focused on combined associations. Previous studies have investigated the interaction between dietary patterns and physical activity for BP and report that both may attribute to more optimal BP control (32, 33), while a superior effect is observed when combining diet and PA (34). However, other studies have provided inconsistent results that adding exercise to diet has no further effect on reducing BP (35).

Therefore, the purpose of this study was to investigate the associations between BP with dairy consumption and PA in a sample of Chinese young women. We hypothesized that high levels of dairy consumption and PA are independently beneficial for BP in this population. We also hypothesized that dairy intake and PA had a combined effect on healthy BP.

## Materials and methods

### Study design and participants

**FIGURE 1:**
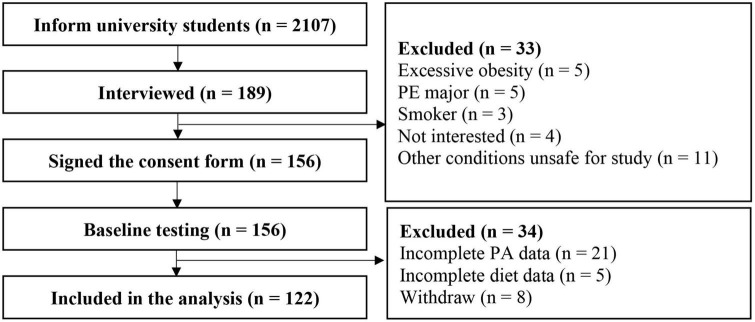
The process of sample. PA, physical activity; PE, physical education.

This cross-sectional study was based on baseline data from a 12-week randomized controlled trial which investigated the effects of high-intensity interval training on cardiometabolic health and PA in female university students. Female university students in the Physical Fitness in Campus (PFIC) study were invited *via* mobile messages and WeChat groups. The PFIC study was a prospective study of 3,829 students (2,107 females and 1,722 males) from Ningbo University who were enrolled in September 2021. The PFIC study was designed to investigate changes in health-related physical fitness and to explore potential risk factors during campus life. Students underwent health-related physical fitness tests once a year during their 4 years of college, including body composition, cardiorespiratory fitness, flexibility, muscular endurance, and muscular strength. Introductions of the exercise intervention study and the cross-sectional study were presented during weekly PE classes. During the introduction, we briefly described the project background, training requirements (30 min per session, 3 sessions per week for a total of 12 weeks), and the major measurements (body composition, aerobic capacity, and blood samples). Interested students went through the following procedures individually: a face-to-face interview, a medical examination, a written consent form and a pre-test. Students with diabetes, on a diet, who were pregnant, or had any other conditions that might affect PA and dietary intake were excluded (e.g., physical disability or injury, cardiovascular diseases, cow’s milk protein allergy and lactose intolerance). In addition, students with severe obesity (BMI ≥35 kg/m^2^) were not included in the present study because they were not included in the regular PE classes. Instead, they were instructed to attend a weight loss intervention, which affects their daily diet and PA. Participants were required to attend the laboratory on weekday mornings in March 2022 to complete all measurements. Detailed processes of samples are presented in Figure 1. Finally, 122 female students who had complete dietary, PA, laboratory, lifestyle, and personality data were included in this cross-sectional analysis. The study was approved by the University of Ningbo institutional review board.

### Physical, physiological, and body composition measurement

Participants were instructed to refrain from alcohol and strenuous exercise 24 h prior to the measurements. Height was measured in duplicate, using a standard stadiometer protocol (36). Weight was measured using bioelectrical impedance analysis (MC-180, TANITA Co., Dongguan, China) under the guidance of two trained staff. Body mass index (BMI) was calculated using standardized equations.

### Blood pressure

Systolic blood pressure (SBP) and diastolic blood pressure (DBP) were measured by trained research staff using an automatic upper arm sphygmomanometer (HEM-1000, Omron, China). Prior to BP measurement, participants were required to sit and rest for at least 5 min. BP measurements were taken in a seated position from the left arm, with the upper section of the arm supported at the heart level. Three measurements were performed at 1-min intervals and the average of the second and the third readings were used for analysis. If the two readings differed by more than 5 mm Hg, an additional measurement was taken. BP was classified based on the recommendation from the American Heart Association as normal (SBP < 120 mm Hg and DBP < 80 mm Hg), elevated (SBP = 120–129 mm Hg and DBP < 80 mm Hg), HTN stage 1 (SBP = 130–139 mm Hg or DBP = 80 – 89 mm Hg), and HTN stage 2 (SBP ≥ 140 mm Hg or DBP ≥ 90 mm Hg). Participants with elevated, HTN stage 1 or stage 2 were identified as having unhealthy BP profiles.

### Dietary intake

Dietary intake was assessed by a staff-administered semi-quantitative food frequency questionnaire (FFQ). This 127-item FFQ was modified from the validated questionnaire used in the 2002 China Nutrition and Health Survey (37). Dairy products included fluid milk, milk power, and yogurt. The consumption frequency included: (1) never, (2) less than once per month, (3) 1–3 times per month, (4) once per week, (5) 2–3 times per week, (6) 4–5 times per week, (7) once per day, (8) twice per day, and (9) three or more times per day. The consumption quantity of fluid milk and yogurt was recorded as serving with the serving size consisting of 250 g and 250 g, respectively. Milk powder was recorded in grams, and 40 g milk power was estimated as a single serving. Samples were presented to help participants to more accurately record serving sizes. Total dairy intake was calculated as the sum of these three categories. Other dietary data were recorded, and the intake of nutrients were calculated according to the Chinese Food Composition Tables (38) and manufacturer information.

### Physical activity

Physical activity was measured using a triaxial accelerometer (ActiGraph, wGT3X-BT, Pensacola, FL, USA), which is a valid measure for university students (39). Participants were instructed to wear the accelerometer on the non-dominant hip for 7 consecutive days except during water-based activities (40). A valid day was defined as not less than 75% of the wear time between 7 a.m. to 11 p.m. and participants provided at least 4 valid days including at least one weekend included in the analysis. The intensity of PA was classified according to the Freedson Adult algorithm. Sedentary was defined as < 100 counts per minute (cpm), light intensity physical activity (LPA) was defined as 100–1,951 cpm, moderate intensity physical activity (MPA) was defined as 1,952–5,724 cpm, vigorous intensity physical activity (VPA) as >5,725 cpm, and moderate-to-vigorous intensity physical activity (MVPA) as >1,952 cpm. Total physical activity (TPA) was defined as the daily vector magnitude cpm.

### Personality

Previous studies have reported that personality is associated with BP recordings (41, 42). Furthermore, studies have shown associations between personality with PA (43, 44) and dietary intake (45, 46). In the present study, both regression analysis with controlled and non-controlled personality data was provided. The “Big Five” dimensions of extraversion, neuroticism, conscientiousness, openness, and agreeableness have been commonly used as measures of personality. In this study, the Chinese Big Five Personality Inventory Shortened Version (CBF-PI-15) was used and has been proven to be a valid and reliable informative alternative when personality is not the main purpose of the study (47). The CBF-PI-15 consisted of 15 items, with 3 items to measure each personality dimension.

### Other variables

Other variables including demographic data, lifestyle and family history of hypertension were identified using a standardized questionnaire. Smoking, drinking, and staying up late were classified as never, sometimes, or always. The family history of hypertension was classified as yes or no.

### Statistical analysis

The sample size was estimated *a priori* using G*Power (version 3.1.9.7) (Heinrich Heine University, Dusseldorf, Germany) under a F tests linear multiple regression designs for fixed models with R^2^ increase. The effect size f^2^, power, and alpha were set at 0.15, 0.95, and 0.05, respectively. With 3 tested predictors and the total of 18 predictors, a total of 120 participants were required.

Descriptive analyses were summarized as Means + SD and proportions for continuous and categorical variables. Normality was checked using the Kolmogorov–Smirnov test. ANOVA and Fisher’s exact test were used to analyze differences between continuous and categorical variables, respectively. Least significant difference or Bonferroni *post hoc* test was used to detect any differences between groups. Correlations between variables were examined using the Pearson product moment correlation coefficient. Linear regression models were used to test the association between dairy consumption (servings per day), PA (MVPA and TPA), and BP (SBP and DBP). The variance inflation factor (VIF) was used to check the collinearity. The z-score method was used to standardize variables. For SBP and DBP, three models were used. The independent variable was dairy consumption in model 1, dairy consumption and MVPA in model 2, and dairy consumption, MVPA and TPA in model 3. In model 2 and model 3, the interaction terms (dairy consumption × MVPA for model 2, dairy consumption × MVPA and dairy consumption × TPA for model 3) were estimated to explore the combined effects. Additionally, we assessed interaction by the BP groups. If there was a statistically significant interaction, separate regression would be performed for healthy and unhealthy BP groups. All models controlled for age, alcohol, smoking, staying up late, family history of hypertension, BMI, total calorie intake, carbohydrate, fat intake (percentage of total calories), and dietary fiber intake (gram). Furthermore, for all models, we further controlled for personality data. Statistical analyses were performed using SPSS for windows, version 23.0 (Chicago, IL, USA) and the significance level was set as *p* < 0.05.

## Results

The characteristics of the participants according to BP ranges are presented in Table 1. Most participants had normal BP (41.80%, *n* = 51), with 23.77% (*n* = 29) classified as elevated, 29.51% (*n* = 36) as HTN stage 1 and 4.92% (*n* = 6) as HTN stage 2. There were no statistically significant differences in age (*p* = 0.889), BMI (*p* = 0.407), alcohol (*p* = 0.976), smoking (*p* = 1.000), staying up late (*p* = 0.775), family history of hypertension (*p* = 0.087), personality score of conscientiousness (*p* = 0.296), agreeableness (*p* = 0.956), openness (*p* = 0.888), and extraversion (*p* = 0.870) between each category. Participants with normal BP had significantly lower scores for neuroticism and were less likely to have a family history of HTN than those with unhealthy BP.

**TABLE 1:** Characteristics of participants.

	All (*n* = 122)	Normal (*n* = 51, 41.80%)	Elevated (*n* = 29, 23.77%)	HTN Stage 1 (*n* = 36, 29.51%)	HTN Stage 2 (*n* = 6, 4.92%)	*p*-value
Age (years)	20.4 ± 1.56	20.38 ± 1.40	20.49 ± 1.49	20.41 ± 1.24	20.01 ± 0.98	0.889
BMI (kg/m2)	20.66 ± 2.58	20.59 ± 2.83	21.00 ± 2.64	20.26 ± 2.27	21.96 ± 1.39	0.407
Alcohol						0.976
Never	105 (86.07%)	43 (84.31%)	25 (86.21%)	31 (86.11%)	6 (100%)	
Sometimes	17 (13.93%)	8 (15.69%)	4 (13.79%)	5 (13.89%)	0 (0.00%)	
**Always**						
Smoking						–
Never	122 (100.00%)	51 (100%)	29 (100%)	36 (100%)	6 (100%)	
Sometimes						
**Always**						
Staying up late						0.775
Never	0	0	0	0	0	
Sometimes	27 (22.13%)	11 (21.57%)	5 (17.24%)	9 (25.00%)	2 (33.33%)	
Always	95 (77.87%)	40 (78.43%)	24 (82.76%)	27 (75.00%)	4 (66.67%)	
Family history of hypertension						0.087
Yes	9 (7.38%)	1 (1.96%)	2 (6.90%)	5 (16.13%)	1 (16.67%)	
No	113 (92.62%)	50 (98.04%)	27 (93.1%)	31 (83.87%)	5 (83.33%)	
**Personality**						
Neuroticism	10.61 ± 3.06	9.57 ± 2.77	10.32 ± 2.27	11.66 ± 1.99	13.17 ± 1.33	<0.001
Conscientiousness	13.5 ± 1.71	13.57 ± 1.76	13.86 ± 1.73	13.11 ± 1.60	12.69 ± 1.70	0.296
Agreeableness	14.82 ± 2.15	14.71 ± 2.36	14.90 ± 2.11	14.88 ± 1.96	14.63 ± 1.15	0.956
Openness	9.91 ± 2.83	9.71 ± 2.74	10.19 ± 2.86	9.93 ± 2.97	10.28 ± 3.03	0.888
Extraversion	9.35 ± 3.44	9.55 ± 3.53	9.40 ± 3.29	9.18 ± 3.48	8.33 ± 3.94	0.87
Energy intake (kcal/day)	1,679.88 ± 518.17	1,599.25 ± 456.28	1,632.26 ± 462.69	1,739.93 ± 591.94	2,235.19 ± 537.24	0.029
Carbohydrate (% in energy)	46.99 ± 4.71	47.14 ± 3.92	46.24 ± 4.28	47.58 ± 5.53	45.81 ± 7.61	0.633
Fat (% in energy)	36.44 ± 3.02	35.5 ± 2.78	36.54 ± 2.48	37.15 ± 3.32	39.70 ± 2.42	0.002
Fiber (g/d)	14.01 ± 5.03	15.22 ± 4.57	13.08 ± 6.24	12.98 ± 4.56	14.48 ± 3.49	0.138
Dairy intake (serving/d)	1.20 ± 0.54	1.42 ± 0.53	1.07 ± 0.53	1.06 ± 0.45	0.74 ± 0.50	0.001
MVPA (min/d)	103.20 ± 16.73	110.00 ± 14.06	99.82 ± 17.41	96.16 ± 16.39	95.85 ± 10.58	<0.001
TPA (cpm)	1,192.31 ± 252.59	1,291.87 ± 231.26	1,203.36 ± 272.97	1,082.02 ± 213.94	954.36 ± 119.21	<0.001
SBP (mm Hg)	121.89 ± 11.39	110.86 ± 5.66	124.29 ± 2.76	132.00 ± 5.59	143.27 ± 2.52	<0.001
DBP (mm Hg)	70.35 ± 6.24	68.57 ± 5.57	68.96 ± 4.02	73.24 ± 7.49	75.62 ± 3.32	<0.001

BMI, body mass index; cpm, count per minute; DBP, diastolic blood pressure; HTN, hypertension; MVPA, moderate-to-vigorous intensity physical activity; SBP, systolic blood pressure; TPA, total physical activity.

The average SBP for all participants was 122 ± 11 mm Hg. For the normal, elevated, HTN stage 1 and HTN stage 2 groups, the average SBP was 111 ± 6, 124 ± 3, 132 ± 6, and 143 ± 3 mm Hg, respectively, and all values were significantly different from the others (*p* < 0.001) The average DBP for all samples was 70 ± 6 mm Hg, and the normal (69 ± 6 mm Hg) and elevated (69 ± 4 mm Hg) group had significant lower values than the HTN stage 1 (73 ± 7 mm Hg, *p* < 0.001 for normal group and *p* = 0.004 for elevated group) and stage 2 (76 ± 3 mm Hg, *p* = 0.006 for normal group and *p* = 0.011 for elevated group) group.

For average daily dietary consumption, there were no statistically significant differences between groups in percentage of energy intake of carbohydrate (46.99 ± 4.71%, *p* = 0.633) and fiber (14.01 ± 5.03 g, *p* = 0.138). The HTN stage 2 group had significantly higher total calories (2,235.19 ± 537.24 kcal/day, *p* = 0.029) and percentage of fat (39.70 ± 2.42%, *p* = 0.020) intake compared to other groups. The average daily dairy consumption was 1.20 ± 0.54 serving per day, which was significantly higher in the normal group (1.42 ± 0.53 serving per day, *p* = 0.001).

The average daily MVPA and TPA were 103.20 ± 16.73 min/d and 1,192.31 ± 252.59 cpm, respectively. There were statistically significant differences in both MVPA (*p* < 0.001) and TPA (*p* < 0.001) between groups and those in the normal group engaged in more MVPA and TPA per day.

Furthermore, we compared healthy and unhealthy BP groups (Table 2) and found statistically significant differences in SBP (*p* < 0.001), DBP (*p* = 0.007), the score of neuroticisms (*p* < 0.001), intake of fat (*p* = 0.003), fiber (*p* = 0.024) and dairy products (*p* < 0.001), MVPA (*p* < 0.001) and TPA (*p* < 0.001). Correlation coefficients between dietary, PA, and BP measures are presented in Table 3. SBP and DBP were significantly correlated (*r* = 0.25, *p* < 0.01). SBP was negatively associated with daily consumption of fiber (*r* = −0.21, *p* < 0.05) and dairy (*r* = −0.41, *p* < 0.01), MVPA (*r* = −0.37, *p* < 0.01), and TPA (*r* = −0.41, *p* < 0.01), whereas these relationships were not significant with DBP. Daily percentage intake of fat and the score for neuroticism were significantly associated with SBP (*r* = 0.27, *p* < 0.01 for fat; *r* = 0.485, *p* < 0.01 for neuroticism) and DBP (*r* = 0.26, *p* < 0.01 for fat; *r* = 0.308, *p* < 0.01 for neuroticism). Furthermore, there was a significant relationship between MVPA and TPA (*r* = 0.41, *p* < 0.01).

**TABLE 2:** Characteristics of participants with healthy or unhealthy blood pressure.

	All (*n* = 122)	Healthy (*n* = 51)	Unhealthy (*n* = 71)	*P*-value
Age (years)	20.4 ± 1.56	20.38 ± 1.40	20.41 ± 1.32	0.911
BMI (kg/m2)	20.66 ± 2.58	20.59 ± 2.83	20.71 ± 2.40	0.808
Alcohol				0.792
Never	105 (86.07%)	43 (84.31%)	62 (87.32%)	
Sometimes	17 (13.93%)	8 (15.69%)	9 (12.68%)	
**Always**				
Smoking				–
Never	122 (100.00%)	51 (100%)	71 (100%)	
**Sometimes**				
**Always**				
Staying up late				0.899
**Never**				
Sometimes	27 (22.13%)	11 (21.57%)	16 (22.54%)	
Always	95 (77.87%)	40 (78.43%)	55 (77.46%)	
Family history of hypertension				0.078
Yes	9 (7.38%)	1 (1.96%)	8 (11.27%)	
No	113 (92.62%)	50 (98.04%)	63 (88.73%)	
**Personality**				
Neuroticism	10.61 ± 3.06	9.53 ± 2.73	11.27 ± 2.22	<0.001
Conscientiousness	13.29 ± 1.78	13.57 ± 1.76	13.45 ± 1.68	0.708
Agreeableness	14.57 ± 2.18	14.71 ± 2.36	14.90 ± 1.99	0.622
Openness	10.04 ± 3.02	9.71 ± 2.74	10.06 ± 2.90	0.502
Extraversion	9.39 ± 3.63	9.55 ± 3.53	9.21 ± 3.38	0.595
Energy intake (kcal/day)	1,679.88 ± 518.17	1,599.25 ± 456.28	1,737.81 ± 554.32	0.146
Carbohydrate (% in energy)	46.99 ± 4.71	47.14 ± 3.92	46.88 ± 5.22	0.767
Fat (% in energy)	36.44 ± 3.02	35.5 ± 2.78	37.12 ± 3.02	0.003
Fiber (g/d)	14.01 ± 5.03	15.22 ± 4.57	13.15 ± 5.20	0.024
Dairy intake (serving/d)	1.20 ± 0.54	1.42 ± 0.53	1.04 ± 0.49	<0.001
MVPA (min/d)	103.2 ± 16.73	110.00 ± 14.06	97.63 ± 16.34	<0.001
TPA (cpm)	1,192.31 ± 252.59	1,291.87 ± 231.26	1,120.79 ± 244.29	<0.001
SBP (mm Hg)	121.89 ± 11.39	110.86 ± 5.66	129.82 ± 7.01	<0.001
DBP (mm Hg)	70.35 ± 6.24	68.57 ± 5.57	71.63 ± 6.42	0.007

BMI, body mass index; cpm, count per minute; DBP, diastolic blood pressure; HTN, hypertension; MVPA, moderate-to-vigorous intensity physical activity; SBP, systolic blood pressure; TPA, total physical activity.

**TABLE 3:** Correlation coefficients between dietary, PA, and BP measures.

	Energy intake	Carbohydrate	Fat	Fiber	Dairy	MVPA	TPA	SBP	DBP
Energy intake	1.00	-	-	-	-	-	-	-	-
Carbohydrate	-0.06	1.00	-	-	-	-	-	-	-
Fat	0.14	-0.08	1.00	-	-	-	-	-	-
Fiber	0.03	-0.06	-0.11	1.00	-	-	-	-	-
Dairy	0.17	0.01	-0.10	-0.05	1.00	-	-	-	-
MVPA	0.08	-0.07	-0.07	0.14	0.17	1.00	-	-	-
TPA	0.05	-0.17	-0.04	0.04	0.21*	0.41**	1.00	-	-
SBP	0.15	-0.04	0.27**	-0.21*	-0.41**	-0.37**	-0.41**	1.00	-
DBP	0.03	0.19*	0.26**	-0.01	-0.11	-0.14	-0.12	0.25**	1.00

DBP, diastolic blood pressure; MVPA, moderate-to-vigorous intensity physical activity; SBP, systolic blood pressure; TPA, total physical activity. *p < 0.05; **p < 0.01.

Regression analysis after controlling for potential variables is summarized in Table 4. In Model 1 (*R*^2^ = 0.344), dairy intake was significantly associated with SBP [standardized beta (b) = −0.425, *p* < 0.001]. This relationship was attenuated after adding MVPA to model 1. In Model 2 (*R*^2^ = 0.413), dairy intake (*b* = −0.384, *p* < 0.001) and MVPA (*b* = −0.276, *p* < 0.001) were significantly and independently associated with SBP. No interaction between dairy intake and MVPA on SBP was observed (*p* = 0.209). In Model 3 (*R*^2^ = 0.467), there were significant association between dairy intake (*b* = −0.343, *p* < 0.001), MVPA (*b* = −0.172, *p* = 0.033), and TPA (*b* = −0.267, *p* = 0.001) with SBP. Dairy intake and MVPA had no interaction on SBP (*p* = 0.742), nor did dairy intake and TPA (*p* = 0.352).

**TABLE 4:** Standardized regression coefficients of dairy intake, MVPA, and TPA for BP measures (without controlling for personality data).

Dependent variable		Independent variable	Standardized beta	*p*-value
SBP	Model 1: *R*^2^ = 0.344	Dairy intake	−0.425	<0.001
	Model 2: *R*^2^ = 0.413	Dairy intake	−0.384	<0.001
		MVPA	−0.276	<0.001
	Model 3: *R*^2^ = 0.467	Dairy intake	−0.343	<0.001
		MVPA	−0.172	0.033
		TPA	−0.267	0.001
DBP	Model 1: *R*^2^ = 0.178	Dairy intake	−0.068	0.449
	Model 2: *R*^2^ = 0.183	Dairy intake	−0.056	0.535
		MVPA	−0.078	0.392
	Model 3: *R*^2^ = 0.184	Dairy intake	−0.051	0.578
		MVPA	−0.066	0.509
		TPA	−0.032	0.749

DBP, diastolic blood pressure; MVPA, moderate-to-vigorous intensity physical activity; SBP, systolic blood pressure; TPA, total physical activity. All models are adjusted for age, alcohol, smoking, staying up late, family history of hypertension, BMI, total calorie intake, carbohydrate, fat intake (percentage of total calories), and dietary fiber intake (gram).

To eliminate the bias induced by personality, we further controlled for personality data and the results are outlined in Table 5. In Model 1, after controlling for personality data and other potential variables, dairy intake was independently associated with SBP (*b* = −0.342, *p* < 0.001), accounting for 10.2% of the variation. While this relationship was slightly attenuated (*b* = −0.306, *p* < 0.001) when MVPA was added in Model 2, which significantly increased the explained variation in SBP to 53.2% (*R*^2^ = 0.532). In Model 2, MVPA was independently associated with SBP (*b* = −0.259, *p* < 0.001), explaining 5.9% of the variance in SBP. There was no interaction between dairy intake and MVPA on SBP (*p* = 0.636). Moreover, the relationships between SBP with dairy (*b* = −0.275, *p* < 0.001) and MVPA (*b* = −0.167, *p* = 0.027) were both attenuated when TPA (*b* = −0.233, *p* = 0.002) was added in Model 3. TPA significantly further increased the explained variation in SBP with 4.0% (*R*^2^ = 0.572). Dairy intake and MVPA had no interaction on SBP (*p* = 0.732), nor did dairy intake and TPA (*p* = 0.446).

**TABLE 5:** Standardized regression coefficients of dairy intake, MVPA, and TPA for BP measures.

Dependent variable		Independent variable	Standardized beta	*p*-value
SBP	Model 1: *R*^2^ = 0.473	Dairy intake	−0.342	<0.001
	Model 2: *R*^2^ = 0.532	Dairy intake	−0.306	<0.001
		MVPA	−0.259	<0.001
	Model 3: *R*^2^ = 0.572	Dairy intake	−0.275	<0.001
		MVPA	−0.167	0.027
		TPA	−0.233	0.002
DBP	Model 1: *R*^2^ = 0.272	Dairy intake	−0.001	0.993
	Model 2: *R*^2^ = 0.274	Dairy intake	0.006	0.948
		MVPA	−0.048	0.590
	Model 3: *R*^2^ = 0.274	Dairy intake	0.006	0.946
		MVPA	−0.047	0.630
		TPA	−0.002	0.980

DBP, diastolic blood pressure; MVPA, moderate-to-vigorous intensity physical activity; SBP, systolic blood pressure; TPA, total physical activity. All models are adjusted for age, alcohol, smoking, staying up late, family history of hypertension, BMI, personality (neuroticism, conscientiousness, agreeableness, openness, extraversion), total calorie intake, carbohydrate, fat intake (percentage of total calories), and dietary fiber intake (gram).

**FIGURE 2:**
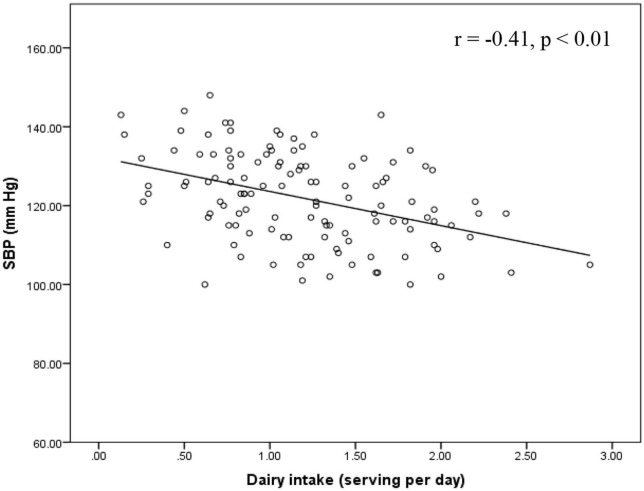
Relationship between SBP and dairy intake in the total sample. SBP, systolic blood pressure.

**FIGURE 3:**
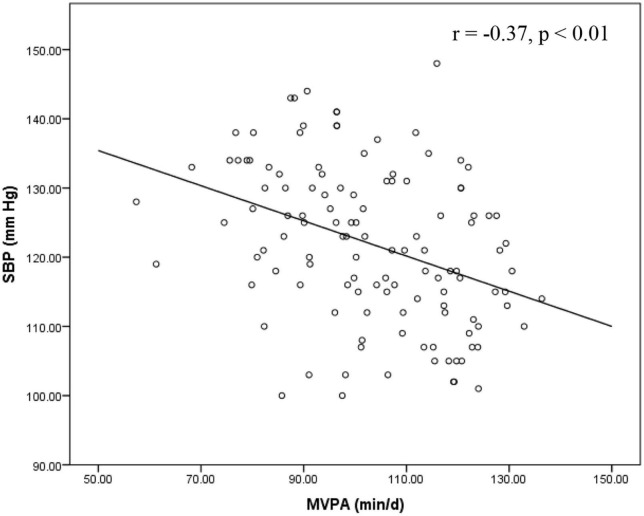
Relationship between SBP and MVPA in the total sample. MVPA, moderate-to-vigorous intensity physical activity; SBP, systolic blood pressure.

**FIGURE 4:**
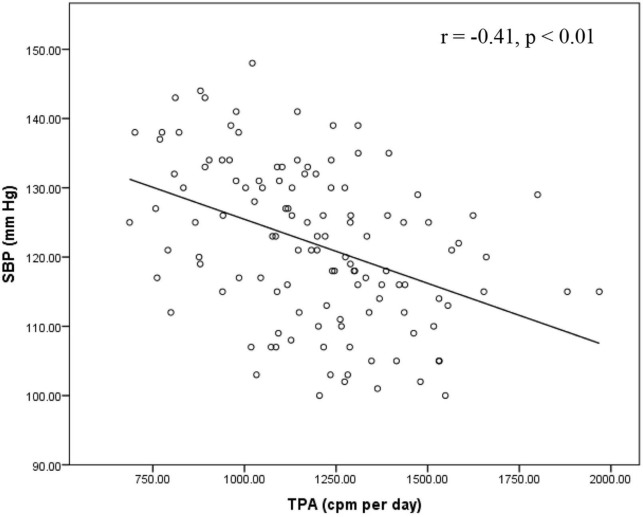
Relationship between SBP and TPA in the total sample. cpm, count per minute; SBP, systolic blood pressure; TPA, total physical activity.

In all models, DBP was not significantly associated with dairy intake, MVPA or TPA in the total sample. There was no interaction between dairy intake with MVPA or TPA on DBP. No interactions were found between BP groups and any exposures (dairy intake, MVPA and TPA) on DBP or SBP. Relationships between dairy intake, MVPA and TPA with SBP are shown in Figures 2–4. For the total samples, each additional serving of dairy, 10 min of MVPA, and 100 cpm of TPA per day, we observed a lower of 5.82 ± 2.94, 1.13 ± 1.01, and 1.10 ± 0.60 mm Hg in SBP, respectively (Figures 2–4).

## Discussion

This study is the first to examine the relationship between dairy intake and PA in relation to BP in a sample of Chinese young women. In our study, approximately 58.2% of young women had unhealthy BP. Our results demonstrated that an average daily dairy serving, MVPA, and TPA were all significantly and independently associated with SBP but not with DBP in Chinese young women. However, our hypothesis that dairy intake and PA had a combined effect on BP was not supported. Overall, dairy intake explained a higher proportion of SBP variance than MVPA and TPA. It indicated that dairy consumption, the intensity, and total volume of PA play important roles in determining BP.

Emerging evidence has revealed that dairy intake is beneficial to BP because dairy products have been shown to improve nitric oxide bioavailability and decrease oxidative stress and inflammation, which have protective effects on vascular function (48). In our study, the average daily dairy intake was 1.20 ± 0.54 per serving. Despite being below the recommended level, dairy intake was associated with a favorable SBP in Chinese young women. This finding has been supported by previous studies. Lin et al. (49) assessed associations between adherence to DASH and BP and reported that dairy intake was only significantly and negatively associated with the change of SBP from the year 1989 to 2002. Likewise, findings from the Guangzhou Biobank Cohort Studies show that the average SBP and DBP were 2.56 mm Hg and 1.32 mm Hg lower in adults who consumed 3 milk servings or more per week compared with those of non-consumers (21). Similar results were reported by Zong et al. (50) who observed that among participants with a median dairy intake of 0.89 serving per day, those who consumed more than 1 serving per day had a 3.54 mm Hg and 2.08 mm Hg lower SBP and DBP than non-consumers. However, this favorable association was not detected with DBP in the current study. The family history of hypertension and percentage daily energy intake of fat and carbohydrate yielded significant but weak associations with DBP.

In agreement with previous studies (22, 23, 25, 51, 52), our results demonstrated that both MVPA and TPA, measured by accelerometers, was significantly related to SBP. This was supported by a previous study in China. Analyzing data from 7,140 adult women who completed at least one of the China Nutrition and Health Survey in 1997, 2000, 2004, or 2006, Chen et al. (53) reported an inverse relationship between MVPA with SBP and DBP in women. Furthermore, we observed an additional 10 min of MVPA per day was associated with 1.13 ± 1.01 mm Hg lower SBP in the total sample. A previous systematic review and meta-analysis examined a 16-week exercise protocol of 120 min of MVPA per week (54). The authors reported significantly pooled reductions in SBP among normotensive, pre-hypertensive, and hypertensive subjects, with a decrease of 0.8 ± 1.4 mm Hg, 4.3 ± 3.4 mm Hg, and 8.3 ± 2.3 mm Hg in SBP, respectively. It seemed that increasing MVPA lowered SBP more in individuals with higher SBP. However, in the present study, BP status had no impact on the association between MVPA and SBP. It indicated that, for a given amount of MVPA, we did not observe lower SBP for participants with hypertension than normotensive participants. This might be due the fact that MVPA was already at a high level in our samples. Conversely, some cross-sectional studies investigating young women did not find any relationship between MVPA and BP. Green et al. (27) examined associations between accelerometer measured PA and cardiometabolic indicators in young women and found that there were no associations between MVPA with BP. Similar results were reported by Slater et al. (29). Moreover, a previous study investigated the effects of meeting PA recommendations on CVD risk factors in young Hispanic women. The findings suggested that there were no differences in BP between women who engaged in at least 30 min of MVPA per day and those who did not (26). One of the potential explanations of the inconsistent results with our study was the normal resting BP status reported in these studies, which has been suggested to influence the magnitude of BP reduction following exercise training (55). In our study, the average SBP recorded was borderline (121.89 ± 11.39 mm Hg), whereas the average DBP was normal (70.35 ± 6.24 mm Hg). BP in pre-hypertensive adults benefited more in response to PA than in normotensive subjects (56, 57). This partially explains why no relationship between DBP and MVPA was detected in our study.

Although PA performed at moderate-to-vigorous intensity was evidenced and recommended to lower BP, findings from our study indicated that TPA was favorably associated with SBP, independent of MVPA. Our finding was supported by a previous meta-analysis. The authors investigated a dose-response relationship between TPA and the incidence of hypertension and reported that normotensive adults who performed 10 metabolic equivalents of task per hour per week had a 6% lower risk of hypertension (58). Few studies have examined the relationship between BP and the device-measuring TPA. Conversely, the study by Slater et al. (29) showed that there was no association between TPA and BP in young normotensive women, whose average daily TPA was 598–731 cpm. Likewise, no association between BP and TPA was reported in women in previous studies that used subjective measures to determine PA (59, 60). The significant association between TPA and SBP in the current study was mainly due to the elevated SBP and high levels of TPA in our sample. Despite the weak but significant correlation, this finding might have important implications for PA strategies to reduce BP. Compared to MVPA, accumulating more TPA appeared to be a better alternative for women who were physically inactive.

Furthermore, our results show that dairy intake, MVPA and TPA were all independently associated with SBP, with dairy intake having the strongest association and MVPA having the weakest. This was in line with the recommended lifestyle strategies aiming to delay or prevent the incidence of hypertension (61). These strategies included maintaining a healthy dietary pattern rich in fruits, vegetables, and low-fat dairy products, as well as increasing PA with a structured exercise protocol. However, there was no consensus on the effectiveness of dietary or PA modification in lowering blood pressure. Consistent with our results, Fagard (35) indicated that diet was more effective than exercise in lowering blood pressure. Whereas a recent study shows a superior effect for PA on lowering BP (25). Although a recent review concluded that there was insufficient evidence to determine whether the frequency, intensity and duration of PA impacted its association with BP (57), increasing TPA might have been more advantageous in our study. Other studies suggested a combination strategy of diet and PA (62, 63). While the combined effect of dairy and PA on BP was not observed in the present study. These inconsistent conclusions might be explained by the mediating role of adiposity and cardiorespiratory fitness on the association between BP with diet and PA (27, 64). In our study, we did not find any relationship between BP and adiposity as measured by BMI in the total sample. This might be due to the exclusion of participants with severe obesity in our study resulting in the normal BMI value (BMI = 20.66 ± 2.58) obtained in our sample. Another explanation could be that BMI may not be a good predictor of adiposity. Previous studies revealed that women with normal BMIs and who have excess fatness had higher BPs (65, 66).

Additionally, our study found that personality characteristics played a role in BP, with the personality score of neuroticisms positively associated with SBP values. Neuroticism has been recognized as being composed of several negative facets such as anxiety, anger hostility, and depression and appeared to be the “disease-prone personality” (67). Our results were in line with the study of Sutin et al. (42) who observed that young adults with higher neuroticism had a greater risk of elevated BP. Similarly, it has been reported that high neuroticism was more likely to induce metabolic syndrome in women than in men (68). Although extraversion, conscientiousness, or agreeableness was shown to be beneficial for BP in other studies, these favorable relationships between BP and other personality traits were not observed in our study. Further research should include measures of personality traits to better understand its effects on BP.

The strengths of our study included the sample of Chinese young women who have not been investigated previously in relation to the association between dairy intake, PA, and BP. Moreover, accelerometers were used to eliminate the report bias of PA measures. Several Limitations in the current study should be noted. First, the cross-sectional design prevented the establishment of causal relationships. Although we had extensively controlled for lifestyle, personality and dietary variables, residual confounding factors such as environment, seasonality and stress were not completely ruled out. Second, the self-reported dairy assessment was subject to recall bias. Although we included the most consumed dairy products in Chinese, some increasingly popular sources of dairy were not counted such as butter, cheese, and ice cream. Moreover, calcium, vitamin D, sodium, potassium, magnesium, and other individual micronutrients were not included due to the semi-quantitative design of FFQ. Finally, results of the current study reflected an education bias. University students with relatively high education level might have more health conscious than the general young population.

## Conclusion

The important findings of this study indicate that the higher amounts of dairy intake, MVPA, and TPA were significantly and independently associated with a lower level of SBP in Chinese young women. Dairy intake was identified to have the strongest association with SBP, followed by TPA and MVPA, respectively. Findings from our study suggested that consuming more dairy products, engaging in more TPA and MVPA were all associated with a lower SBP in Chinese young women. Future studies should seek a quantitative assessment tool to capture dairy intakes as well as the related nutrients to complement the evidence for a protective mechanism of dairy products.

## Data availability statement

The original contributions presented in this study are included in the article/supplementary material, further inquiries can be directed to the corresponding author.

## Ethics statement

The studies involving human participants were reviewed and approved by the Ethics Committee of Research Academy of Grand Health, Ningbo University. The patients/participants provided their written informed consent to participate in this study.

## Author contributions

YL, JB, and HW contributed to the conception and design of the study. YL and QW organized the database. YL and SY performed the statistical analysis. YL wrote the first draft of the manuscript. YL, QW, and SY wrote sections of the manuscript. All authors contributed to the manuscript revision, read, and approved the submitted version.
